# Tannin and Iron-Reactive Phenolics Content in Red Cold-Hardy Hybrid Grape Tissues throughout Development and Ripening

**DOI:** 10.3390/foods13070986

**Published:** 2024-03-23

**Authors:** Alexander D. Gapinski, Nicolas Delchier, Aude A. Watrelot

**Affiliations:** Department of Food Science and Human Nutrition, Iowa State University, 536 Farm House Lane, Ames, IA 50011-1054, USA; gapin2@iastate.edu (A.D.G.); delchier@iastate.edu (N.D.)

**Keywords:** grape skins, grape seeds, ‘Crimson Pearl’, ‘Petite Pearl’, ‘Marquette’, berry growth

## Abstract

Phenolic compounds, especially tannins, are important for red wine quality. Wines made from cold-hardy hybrid grape cultivars have much lower tannin concentrations than wines from *Vitis vinifera* grape cultivars. This study assessed the phenolics content of berry tissues of three red cold-hardy hybrid cultivars in comparison to *V. vinifera* cv. ‘Pinot noir’ throughout development and ripening. Basic chemical properties, iron-reactive phenolics content, and tannin content were evaluated in the juice, skins, and seeds of *Vitis* spp. cvs. ‘Crimson Pearl’, ‘Marquette’, and ‘Petite Pearl’ and ‘Pinot noir’ at six time points from one week post-fruit set to harvest in 2021 and 2022. ‘Crimson Pearl’ displayed similar iron-reactive phenolics and tannin contents in juice, skins (22.6–25.4 mg/g dry skin and 8.0–12.2 mg/g dry skin, respectively), and seeds (12.8–29.8 mg/g dry seed and 4.2–22.0 mg/g dry seed, respectively) as ‘Petite Pearl’ and ‘Marquette’ at harvest in 2022. The hybrid cultivars showed a similar trend of phenolic accumulation as ‘Pinot noir’ but resulted in overall lower content in skins and seeds. Despite differences in developmental trends, the three hybrid grape cultivars displayed similar phenolic content at harvest ripeness. This is the first study examining the phenolic content of ‘Crimson Pearl’ and ‘Petite Pearl’ throughout berry development and ripening. This study provides important information for the wine industry to make informed decisions on making wine with these cultivars.

## 1. Introduction

*Vitis vinifera* is the predominant grapevine species used for quality wine production around the world. Originating in Europe, this species is poorly adapted to growing in overly humid or cold climates, such as those found in the U.S. Midwestern and Northeastern regions. Interspecific cold-hardy grape cultivars are crosses between *V. vinifera* and North American *Vitis* spp. (e.g., *V. riparia*, *V. rotundafolia*, *V. rupestris*, and *V. labrusca*) and are grown in these regions for their cold hardiness and tolerance to pests and disease [1,2]. The most widely planted cold-hardy hybrid red grape cultivars are ‘Marquette’, ‘Frontenac’, ‘Maréchal Foch’, and ‘St. Croix’. However, the wines made from these cultivars often display high acidity, vegetal aromas, low color stability, and low astringency due to low tannin concentrations and therefore lead to lower-quality wines [3,4]. As previously published, red wines with lower tannin and phenolics concentrations scored lower when allocating grades to ‘Shiraz’ and ‘Cabernet Sauvignon’ wines on “fitness for purpose” by senior winemakers [5]. Additionally, Springer and Sacks [6] showed that red wines made from cold-hardy hybrid cultivars exhibited an average of 5.5-fold lower concentrations of tannins than red wines made from *V. vinifera* grapes, which may lead to overall lower red hybrid wine quality.

Phenolic compounds, especially flavonoids, such as condensed tannins (i.e., oligomers or polymers of flavan-3-ols) and anthocyanins, are extracted from grape skins and seeds during winemaking and confer quality markers, including antioxidants, astringency, and color saturation and stability, to red wines [7]. The concentration and structure of these compounds in the wine depends on many factors including the growing environment and cultivars. Previous works on *V. vinifera* grape cultivars, such as ‘Cabernet Sauvignon’ [8,9], ‘Shiraz’/’Syrah’ [10,11], ‘Pinot noir’ [9,12], and ‘Merlot’ [8], illustrated variation in the content of phenolic compounds in grapes and wines. For example, Harbertson et al. [9] found total tannin content ranging from 1.02 to 1.76 mg/berry in ‘Pinot noir’ and 1.18 and 1.92 mg/berry in ‘Cabernet Sauvignon’ and ‘Syrah’, respectively. Even though ‘Syrah’ showed the highest total tannin content per berry, wines made from ‘Cabernet Sauvignon’ were the richest in tannin (594 mg/L compared to 68 to 217 and 246 mg/L in ‘Pinot noir’ and ‘Syrah’, respectively). Another study by Downey and colleagues [11] found a higher total extractable tannin content in ‘Syrah’ (5.71 mg/berry), most likely due to different vintage, location, and environmental conditions. In contrast to *V. vinifera* grapes, the tannin content in the skins and seeds of ‘Maréchal Foch’ and ‘Corot noir’, two hybrid grape cultivars, was found to be 1.8-fold lower [6]. Skin tannin content in hybrids was much lower than in *V. vinifera*, whereas seed tannin content was about the same. However, studies such as these have only compared hybrids and *V. vinifera* at harvest maturity, so it is still not clear how tannin content is impacted during the development and ripening of hybrid grape cultivars. 

Other studies by Downey et al. [11] and Harbertson et al. [9] evaluating tannin content in seeds throughout development and ripening have shown trends that suggest a change in biosynthetic enzyme activity. In both studies, the seeds had a high initial tannin content followed by a decrease observed shortly after fruit set and an increase just before véraison. Despite changes in the timing of tannin accumulation, comparable tannin content was observed at commercial harvest between two years [9]. Nevertheless, the development of skin and seed tannins in *V. vinifera* cultivars leads to controversial information and is dependent on the cultivar, year, and environment. Although the evolution of tannin content in the grape tissues of non-*V. vinifera* cv. ‘Marquette’ throughout berry development and ripening has previously been evaluated [13], it has not been directly compared to *V. vinifera*. Additionally, research on some red cold-hardy hybrids, such as ‘Crimson Pearl’ and ‘Petite Pearl’, is scarce. Studying the development of phenolic compounds in those hybrids will help to illuminate and optimize viticultural and winemaking practices to maximize the quality of red wines made from those cultivars. 

The goal of this study was to compare the basic chemical parameters, tannin, and iron-reactive phenolics content in the grape juice, skins, and seeds of three red cold-hardy hybrid grape cultivars (i.e., ‘Crimson Pearl’, ‘Marquette’, and ‘Petite Pearl’) and *V. vinifera* cv. ‘Pinot noir’ throughout berry development and ripening in 2021 and 2022.

## 2. Materials and Methods

### 2.1. Plant Material

‘Marquette’, ‘Petite Pearl’, and ‘Crimson Pearl’ grapevines were from the Iowa State University (ISU) Horticulture Research Station in Ames, IA, USA (42°06′ N, 93°35′ W, 300 m elevation, plant hardiness zone 5a, Clarion loam soil). ‘Marquette’ was introduced in 2006 by the University of Minnesota and is a cross of two hybrid cultivars, MN 1094 and Ravat 262, which include *V. riparia* and *V. vinifera* in their parentage, respectively. ‘Petite Pearl’ (released in 2009) and ‘Crimson Pearl’ (released in 2015) were both crossed in 1996 from MN 1094 and E. S. 4-7-26 hybrids. The ‘Marquette’ vines were planted in 2011, and the ‘Crimson Pearl’ and ‘Petite Pearl’ vines were planted in 2018. All three cultivars were planted with 2.44 m between vines and 3.05 m between rows, trained on a single high-wire (high cordon) trellis system with north–south row orientation. ‘Pinot noir’ grapevines were from Brys Estate Vineyard in the Old Mission Peninsula American Viticultural Area (AVA), MI, USA (44°53′ N, 85°30′ W, 223 m elevation, plant hardiness zone 6a, sandy and sandy-loam soils). The ‘Pinot noir’ vines were planted in 2002 (rootstock 3309, scion 667, clone 115) with north–south row orientation, 1.22 m between vines and 2.74 m between rows, and trained using vertical shoot positioning (VSP) or double Guyot. 

Healthy grape clusters (unaffected by *Botrytis cinerea*) were sampled from the same vines at six phenological time points throughout development and ripening (1 wk post-fruit set [PFS], 3 wks PFS, 5 wks PFS, véraison, mid-ripening, and harvest) in 2021 and 2022 (Table A1). One cluster of grapes from nine vines (‘Marquette’), one to two clusters from 16 vines (‘Crimson Pearl’ and ‘Petite Pearl’), and a total of 5 to 6 clusters of ‘Pinot noir’ grapes were collected randomly from different parts of the vines. ‘Marquette’ was harvested on 25 August 2021 and 26 August 2022, ‘Petite Pearl’ and ‘Crimson Pearl’ were both harvested on 8 September 2021 and 9 September 2022, and ‘Pinot noir’ was harvested on 7 October 2022. At each time point, clusters from the ISU Horticulture Research Station were transported to the laboratory in a cooler with ice packs within one hour of collection. ‘Pinot noir’ was packed in an insulated box with ice packs and shipped from Michigan to arrive at ISU between 2 and 3 days later and directly processed. The basic chemical parameters are shown in Table 1.

### 2.2. Weather Data

**Figure 1:**
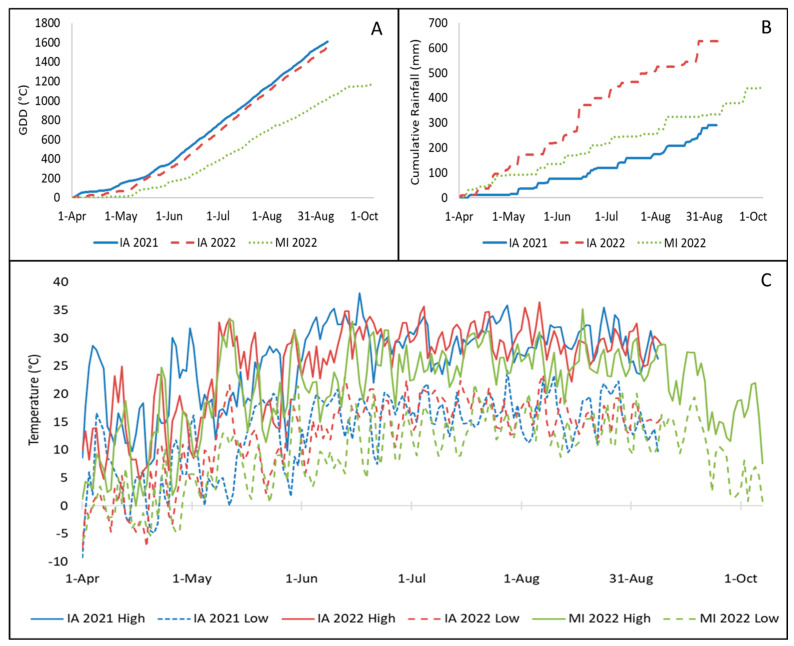
Weather data from 1 April to 9 September (IA) and 7 October (MI). (**A**) Growing Degree Days (GDD) base 10 °C, (**B**) cumulative rainfall (mm), (**C**) daily high and low temperatures (°C) in 2021 and 2022 at the ISU Horticulture Research Station (IA) and in 2022 in Old Mission, Michigan (MI).

Weather data including temperature (°C), Growing Degree Days (GDD, base 10 °C), and precipitation (mm) were recorded for each season from 1 April until harvest for both vineyard locations (Figure 1). Iowa weather data were accessed from the Iowa State University Iowa Environmental Mesonet (https://mesonet.agron.iastate.edu/GIS/apps/agclimate/gsplot.phtml? accessed on 9 August 2023), and Michigan weather data were accessed from the Michigan State University Enviro-weather (formerly Michigan Automated Weather Network [MAWN], https://mawn.geo.msu.edu/station.asp?id=old&rt=24, accessed on 9 August 2023) program.

### 2.3. Chemicals

Potassium metabisulfite (KMBS) and sodium dodecyl sulfate (SDS) were purchased from Acros Organics (Fisher Scientific, Fair Lawn, NJ, USA). Triethanolamine (TEA) was purchased from Aqua Solutions (Deer Park, TX, USA). Glacial acetic acid, acetone, acetonitrile (HPLC grade), D-fructose, ethyl alcohol (200 proof), ferric chloride, hydrochloric acid (37.5%), methanol (HPLC grade), and *ortho*-phosphoric acid were purchased from Fisher Scientific (Fair Lawn, NJ, USA). Sodium hydroxide (0.1 N) was purchased from Lab Chem (Zelienople, PA, USA). (−)-Epicatechin (purity ≥ 90%), (+)-catechin hydrate (purity ≥ 98%), glucose, L-tartaric acid, and trifluoroacetic acid (TFA) were purchased from Sigma-Aldrich (St. Louis, MO, USA).

### 2.4. Preparation of Berries, Juices, Skin, and Seed Samples

One hundred berries from each time point for each cultivar were randomly sampled (once for each time point before harvest and three times at harvest) for juice analysis from the collected clusters. The berries were crushed by hand in a zip-top bag using the heel of the palm for about one minute until all berries had burst and cells had ruptured to exude the maximum volume of juice. The juice was poured off the solids into a labeled tube, and basic chemical properties, total iron-reactive phenolics, and tannin concentration were evaluated the same day, as described in separate, following sections. Fifty berries were randomly sampled (once for each time point before harvest and three times at harvest) for tissue separation from the collected clusters. The weight of the 50 berries was recorded, along with the equatorial diameter (using a caliper halfway between the pedicel end and point of rupture) for 20 randomly selected berries (Table A2). The fifty berries were frozen at −20 °C prior to tissue separation to better manage time of preparation and analysis. To separate the berry tissues, berries were removed from the freezer and kept on ice to peel skins and separate seeds from the thawing flesh using tweezers. The seeds were counted, and the pulp was discarded. Skins and seeds were put in separate tubes, weighed, and stored at −80 °C overnight and then freeze-dried for 24 h using a Labconco FreeZone 2.5 benchtop freeze dryer (Labconco Corporation, Kansas City, MO, USA). The dry weight was recorded after freeze-drying.

### 2.5. Basic Chemical Properties of Juice

Basic chemical parameters including pH, titratable acidity (TA), and °Brixtotal soluble solids (TSS) were measured in juice samples the day of collection or the day after collection (juice for ‘Marquette’ at harvest and ‘Pinot noir’ for all time points was stored at room temperature overnight). The pH was measured using an Orion Star^TM^ A211 Benchtop pH Meter (Thermo Fisher Scientific, Waltham, MA, USA). Five milliliters of juice (diluted with 100 mL of distilled water) were used to measure titratable acidity with 0.1 N sodium hydroxide to an endpoint of pH 8.20 and expressed in tartaric acid equivalents. An RF153 digital refractometer (FLIR Commercial Systems Inc., Nashua, NH, USA) was used to measure °Brix.

### 2.6. Skin and Seed Extracts

Dry skins and seeds were ground using a blade coffee grinder (for seeds, Hamilton Beach Custom Grind^TM^ model 80393F, Hamilton Beach, Glen Allen, VA, USA) or mortar and pestle (for skins) until a consistent powder was achieved. A solution of 70% acetone in water (*v*/*v*) containing 0.05% TFA was added to 100 mg of powder (50 g/L) and was placed for 30 min in a 30 °C ultrasonic bath (Fisherbrand^®^ FB11203, frequency: 80, power: 100), as described by Watrelot and Bouska [14]. Following extraction, the samples were centrifuged (Sorvall Legend X1R, Thermo Scientific, Waltham, MA, USA) for 5 min at 3000× *g*, and the supernatant was filtered using a syringe fitted with a 13 mm, 0.45 µm PTFE filter. The solvent was evaporated overnight at 35 °C from the filtered extracts using a CentriVap^®^ Complete^TM^ (73150 Series, Labconco Corporation, Kansas City, MO, USA), then the extracts were stored at −80 °C before freeze drying (FreeZone 2.5L, Labconco Corporation, Kansas City, MO, USA) for 24 h. 

In 2021, 2.0 mL of model wine (13.0% (*v*/*v*) ethanol, 5.0 g/L tartaric acid, pH 3.5) was added to dry extracts, vortexed, and sonicated for 1 min at the same settings used for extraction. The dissolution procedure was modified in 2022 (1:14 (*v*/*v*) methanol: model wine) due to insoluble material present after drying. First, two 50 µL additions of methanol were added to the dry extracts, with 3 min of sonication (using previously indicated frequency and power settings) following each addition. Then, two separate additions of model wine (400 µL then 1500 µL) were added, with 3 min of sonication following each model wine addition. The solution was vortexed and sonicated for a final 3 min for a total of 15 min. The dissolved extracts were filtered (0.45µm PTFE filters) prior to iron-reactive phenolics and tannin quantification. To make sure the solubilization methods did not impact the data, a subset of ‘Crimson Pearl’ skin and seed samples from both vintages were extracted using both methods (Figure A2).

### 2.7. Total Iron-Reactive Phenolics Content

The previously published assay by Harbertson and Adams [9] was used to measure the total iron-reactive phenolics (IRP) content in all samples. Briefly, 75 µL of juice or solubilized extract (as described above) was added to 800 µL of a sodium dodecyl sulfate/triethanolamine (SDS/TEA) buffer, and the background absorbance at 510 nm was recorded after a 10 min incubation at room temperature. Then, the final absorbance was recorded after 125 µL ferric chloride reagent was added and an additional 10 min incubation at room temperature. The absorbance values were evaluated against a blank using 875 µL of the same buffer before and after the addition of 125 µL of the same ferric chloride solution. Total iron-reactive phenolics contents were expressed as (+)-catechin equivalents based on the external calibration curve.

### 2.8. Tannin Content

Tannin content was quantified using RP-HPLC-DAD (1260 Infinity II, Agilent Technologies, Santa Clara, CA, USA) with a polystyrene divinylbenzene column (PLRP-S, 2.1 × 50 mm, 100 Å, 3 µm, Agilent Technologies, Santa Clara, CA, USA) and a guard column (PRP-1, 3 × 8 mm, Hamilton Co., Reno, NV, USA) of the same material, as previously published [15]. Briefly, the column oven was at 30 °C. Samples were filtered using a 0.45 µm PTFE filter, and 5 µL was injected. Mobile phases were 1.5% (*w*/*w*) 85% *ortho*-phosphoric acid (mobile phase A) and 20% (*v*/*v*) mobile phase A in acetonitrile (mobile phase B) at a flow rate of 0.30 mL/min and a linear gradient as follows: time in min (% B), 0 (14), 12.6 (34), 12.6−13.3 (34),15.1 (70), 15.1−16.8 (70), 19.6 (14), and 19.6−28.0 (14). Tannin quantification was carried out by first drawing a baseline at 0 mAU across the whole chromatogram and then integrating the peak between 16.8 and 19.8 min. Tannin content was expressed as (−)-epicatechin equivalents based on the external calibration curve.

### 2.9. Statistical Analysis

Data were collected as analytical replicates or biological replicates as noted where appropriate. Results were analyzed using one-way analysis of variance (ANOVA) and Tukey’s HSD post-hoc test on JMP 15.2.0 software (SAS Institute Inc., Cary, SC, USA). Statistical significance was determined using α = 0.05 for °BrixTSS, pH, TA, IRP and tannin contents, and berry diameter.

## 3. Results

### 3.1. Weather Data

In Iowa, more growing degree days (base 10 °C, GDD, Figure 1A) and less rainfall (Figure 1B) accumulated early (in April and May) in the 2021 season compared to the 2022 season (1608 GDD and 290 mm in 2021, 1561 GDD and 627 mm in 2022). Fewer GDD (1162) and less rainfall (452 mm) accumulated for the ‘Pinot noir’ vineyard than for the Iowa vineyard in 2022 despite the later October harvest date (Figure 1). The average high and low temperatures in Iowa were higher in 2021 (high: 26 °C, low 13 °C) than in 2022 (high: 25 °C, low 12 °C). The ‘Pinot noir’ vineyard had lower high (21 °C) and low (7 °C) average temperatures than the Iowa vineyard in 2022.

### 3.2. Basic Chemical Properties of Juice

The basic chemical properties of the juice from the ‘Marquette’, ‘Crimson Pearl’, ‘Petite Pearl’, and ‘Pinot noir’ grape cultivars at each phenological time point are summarized in Table 1. Despite a ripening period that was two weeks shorter, degree °Brix was highest in ‘Marquette’ at harvest in both years (26.7 °Brix in 2021 and 22.3 °Brix in 2022). °Brix was higher in ‘Crimson Pearl’ (21.3 °Brix) than in ‘Petite Pearl’ (16.8 °Brix) at harvest in 2021 but was the same (19.0 °Brix) in 2022. °Brix in ‘Marquette’ was especially higher than in ‘Crimson Pearl’ or ‘Petite Pearl’ in 2021, which may be due to a dry period in the middle of August just before ‘Marquette’ was harvested. This dry period was contrasted by rainfall (19.2%/57 mm of the total) in the two weeks leading up to ‘Crimson Pearl’ and ‘Petite Pearl’ harvest in 2021, which may have led to the dilution of soluble solids.

pH was significantly higher for ‘Marquette’ (2.60) than for ‘Crimson Pearl’ (2.49) and ‘Petite Pearl’ (2.47) 1 wk PFS in 2021, but there was no difference in pH between the four cultivars (including ‘Pinot noir’) at this time point in 2022. pH was highest in ‘Marquette’ until around véraison, then pH was lowest in ‘Marquette’ in both years at mid-ripening (2.90 in 2021 and 2.81 in 2022) and harvest (3.01 in 2021 and 2.99 in 2022). TA in ‘Marquette’ was about 1.39 to 2 times higher than any other cultivar at harvest in either year. There was lower TA and higher pH at harvest in ‘Crimson Pearl’ than in ‘Petite Pearl’ in 2021. However, TA and pH did not significantly differ between ‘Crimson Pearl’, ‘Petite Pearl’, and ‘Pinot noir’ in 2022.

### 3.3. Iron-Reactive Phenolics and Tannin Concentrations of Juice

**Figure 2:**
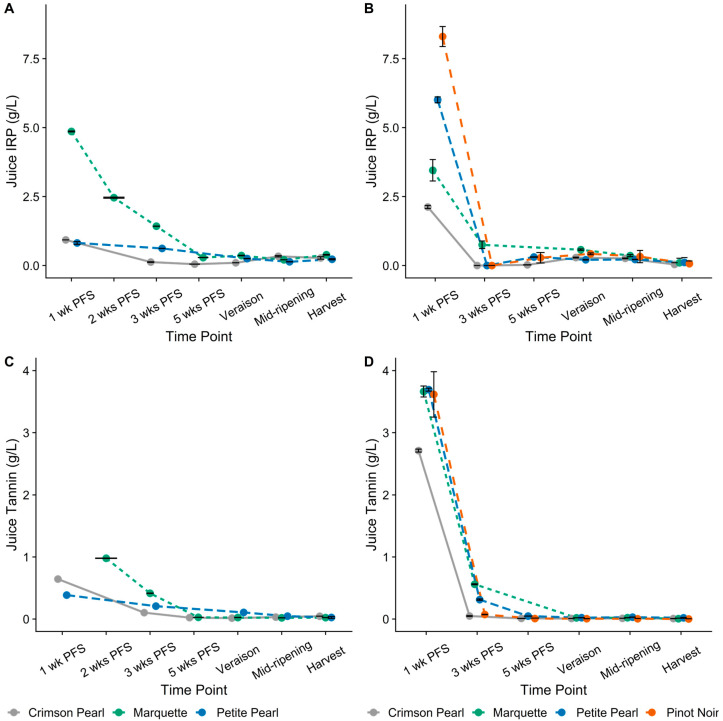
Iron-reactive phenolics (IRP) and tannin concentrations expressed as (+)-catechin equivalents (CE) and (−)-epicatechin equivalents (ECE), respectively, for juice. (**A**) 2021 Juice Tannin, (**B**) 2022 Juice Tannin, (**C**) 2021 Juice IRP, (**D**) 2022 Juice IRP. PFS—post-fruit set. Plotted values show means of three analytical replicates for each time point before harvest; harvest is the mean of three biological replicates. Error bars represent standard deviation. Both line color and line type are unique to each cultivar.

Iron-reactive phenolics (IRP) and tannin concentrations in juices were highest for each cultivar at the beginning of berry development and decreased until harvest (Figure 2). The IRP concentration at 1 wk PFS in 2021 was the highest in ‘Marquette’. At harvest in 2021, the trend was the same with 0.35, 0.28, and 0.23 g/L IRP in ‘Marquette’, ‘Crimson Pearl’, and ‘Petite Pearl’, respectively. In 2022, the highest IRP concentration in juice at 1 wk PFS was observed in ‘Pinot noir’ (8.30 g/L), followed by ‘Petite Pearl’, ‘Marquette’, and ‘Crimson Pearl’ (6.00, 3.45, and 2.12 g/L, respectively). Interestingly, IRP concentration decreased to zero (i.e., <LOQ = 0.13 g/L) after 3 wks PFS in 2022, then recovered slightly at véraison (0.21 to 0.43 g/L IRP) and remained constant until harvest. This pattern was observed in each cultivar except ‘Marquette’, which showed 0.75 g/L IRP at 3 wks PFS. However, spraying berries with a 50 mg/L solution of potassium meta-bisulfite (56% sulfur dioxide [SO_2_]) before crushing to prepare juices prevented this decrease in all cultivars. The bisulfite form of SO_2_ (HSO_3_^−^) is known to bind to enzymes, especially polyphenol oxidase [16], and reduce the risk of enzymatic browning of grape juice, which could explain these observations. Regardless of SO_2_ application, only small differences between cultivars were detected in juice IRP and tannin concentrations after the 3 wk PFS time point, which was most likely due to short contact time between juice, skins, and seeds.

The highest tannin concentration in ‘Marquette’ juices was observed at 2 wks PFS in 2021 (0.98 g/L, not shown in Figure 2), whereas lower juice tannin concentrations were observed in ‘Crimson Pearl’ and ‘Petite Pearl’ at 1 wk PFS (0.64 and 0.39 g/L, respectively, Figure 2A). In 2022, significantly higher tannin concentrations were exhibited in ‘Petite Pearl’ (3.69 g/L), ‘Marquette’ (3.66 g/L), and ‘Pinot noir’ (3.62 g/L) than in ‘Crimson Pearl’ (2.71 g/L) at 1 wk PFS (Figure 2B). At harvest, tannin concentrations in ‘Pinot noir’ and ‘Crimson Pearl’ (0.005 and 0.008 g/L, respectively) were both significantly lower than those in ‘Marquette’ and ‘Petite Pearl’ (0.016 and 0.020 g/L, respectively, Figure 2B). Both IRP and tannin concentrations in juices were higher at 1 wk PFS in 2022 than in 2021 for ‘Crimson Pearl’ and ‘Petite Pearl’.

### 3.4. Phenolics Content of Skins and Seeds

**Figure 3:**
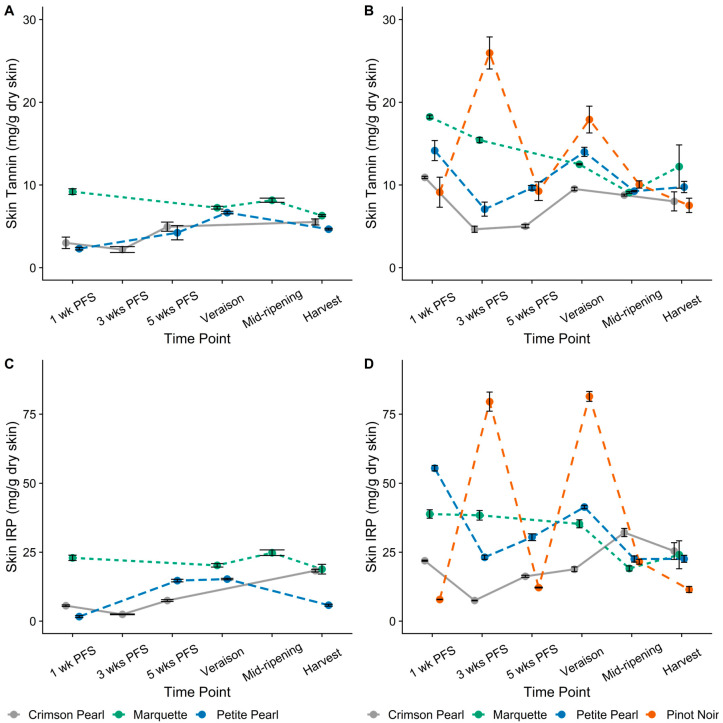
Iron-reactive phenolics (IRP) and tannin contents expressed as (+)-catechin equivalents (CE) and (−)-epicatechin equivalents (ECE), respectively, for skins. (**A**) 2021 Skin Tannin, (**B**) 2022 Skin Tannin, (**C**) 2021 Skin IRP, (**D**) 2022 Skin IRP. PFS—post-fruit set. Plotted values show means of three analytical replicates for each time point before harvest; harvest is the mean of three biological replicates. Error bars represent standard deviation. Both line color and line type are unique to each cultivar.

Skin tannin and IRP contents were highest in ‘Marquette’ at every time point throughout the 2021 growing season except at harvest, when the IRP content of ‘Crimson Pearl’ skins was the same (18.5 mg/g dry skin, Figure 3A,C). The IRP content of ‘Petite Pearl’ skins in 2021 was significantly lower than that of the other two cultivars at harvest, with 6 mg/g dry skin (Figure 3C). In 2022, tannin and IRP contents were higher in skin extracts at the beginning of berry development compared to later time points, as in the juice (Figure 3B,D). At 1 wk PFS, skin IRP content was highest in ‘Petite Pearl’ (55 mg/g dry skin), followed by ‘Marquette’, ‘Crimson Pearl’, and ‘Pinot noir’ (39, 22, and 8 mg/g dry skin, respectively, Figure 3D). However, the highest skin tannin content was observed in ‘Marquette’ (18 mg tannin/g dry skin) at the same time point (Figure 3B). By 3 wks PFS, the highest IRP and tannin contents in skins were observed in ‘Pinot noir’ (80 mg IRP/g dry skin and 26 mg tannin/g dry skin, respectively), followed by ‘Marquette’, ‘Petite Pearl’, and ‘Crimson Pearl’ (38, 23, 7 mg IRP/g dry skin and 15, 7, 5 mg tannin/g dry skin, respectively). From 3 wks PFS until véraison in 2022, skin IRP and tannin contents increased in the hybrid cultivars. This increase was followed by a decline until harvest. Skin IRP and tannin contents decreased in ‘Pinot noir’ from 3 wks to 5 wks PFS, which was followed by an increase at véraison (when it was highest among the four cultivars) and then a decline until harvest, when skin IRP and tannin contents were lowest in ‘Pinot noir’ of any cultivar in 2022.

**Figure 4:**
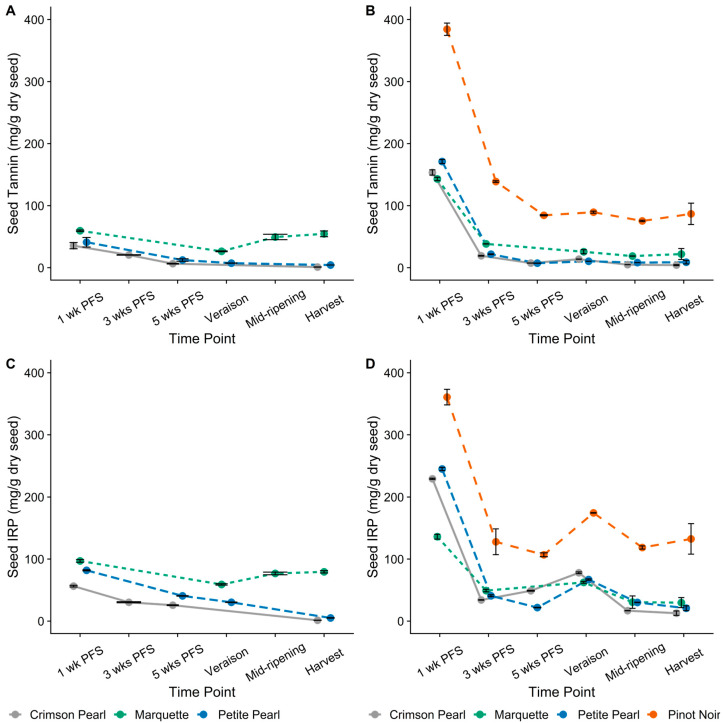
Iron-reactive phenolics (IRP) and tannin contents expressed as (+)-catechin equivalents (CE) and (−)-epicatechin equivalents (ECE), respectively, for seeds. (**A**) 2021 Seed Tannin, (**B**) 2022 Seed Tannin, (**C**) 2021 Seed IRP, (**D**) 2022 Seed IRP. PFS—post-fruit set. Plotted values show means of three analytical replicates for each time point before harvest; harvest is the mean of three biological replicates. Error bars represent standard deviation. Both line color and line type are unique to each cultivar.

Changes in IRP and tannin contents between cultivars were more consistent in seeds compared to skins (Figure 4). In 2021, the highest IRP and tannin contents were maintained in ‘Marquette’ across all time points, followed by ‘Petite Pearl’ and then ‘Crimson Pearl’. IRP and tannin contents in seeds decreased in all cultivars from 1 wk PFS to véraison, when both contents increased in ‘Marquette’ until harvest while continuing to decline in ‘Crimson Pearl’ and ‘Petite Pearl’. At harvest, seed IRP and tannin contents in ‘Marquette’ were 79 mg IRP/g dry seed and 41 mg tannin/g dry seed, whereas they were much lower in ‘Crimson Pearl’ and ‘Petite Pearl’ (1 and 5 mg IRP/g dry seed and 1 and 4 mg tannin/g dry seed, respectively). In 2022, the highest IRP and tannin contents in seeds for all four cultivars were observed at 1 wk PFS. These were much higher (between 40 and 333%) than the same phenological time point in 2021. This high initial seed phenolic content was followed by a decline until, at véraison, an increase was observed, followed by a decline until mid-ripening for all cultivars. This decline continued until harvest in the hybrid cultivar seeds, but IRP content in ‘Pinot noir’ seeds increased. Overall, tannin and IRP contents were higher in ‘Pinot noir’ across all phenological time points in 2022 compared to the hybrid cultivars (Figure 4B,D). At harvest, a seed IRP content of 133 mg IRP/g and tannin content of 87 mg tannin/g dry seed was observed in ‘Pinot noir’, and the IRP and tannin contents in hybrid cultivars ranged from 13 to 30 mg IRP/g and 4 to 22 mg tannin/g dry seed, with the highest in ‘Marquette’, followed by ‘Petite Pearl’ and then ‘Crimson Pearl’.

## 4. Discussion

Although it is known that the trellis system, vine spacing, soil type, and climate have an impact on the phenolics content of grapes, these factors could not be controlled between the hybrid cultivars and ‘Pinot noir’, because *V. vinifera* does not survive the extreme cold encountered during Iowa winters. ‘Pinot noir’ was sourced from Michigan, because it has a similar climate without such cold temperatures. Additionally, ‘Marquette’ has ‘Pinot noir’ in its pedigree. 

The differences in pH and TA between ‘Marquette’ and ‘Petite Pearl’ were in agreement with Scharffeter et al. [17]. In that study, higher pH and lower TA values were observed in ‘Petite Pearl’ than in ‘Marquette’ at harvest over three vintages. A higher TA in ‘Marquette’ compared to ‘Pinot noir’ at harvest was already observed by Teh et al. [18]. In addition to the impact of the cultivar on TA concentration, ‘Marquette’ was harvested 2 weeks before ‘Crimson Pearl’ and ‘Petite Pearl’ in both years and 6 weeks before ‘Pinot noir’ in 2022, which might also explain the difference in concentration. It is worth noting that ‘Crimson Pearl’ and ‘Petite Pearl’ displayed higher pH and lower °Brix and TA values than ‘Marquette’. These observed differences between cultivars may be related to differences in the onset of key phenological stages throughout development and ripening such as acid metabolism and sugar accumulation, thus affecting acidity and °Brix at harvest [2]. Regardless of the underlying cause, lower acidity and °Brix in ‘Crimson Pearl’ and ‘Petite Pearl’ are important aspects for winemakers when considering overall wine quality and balancing sensory attributes. Although ‘Marquette’ is the most widely planted cold-hardy hybrid, the wines are often very sour, which is accentuated by the lack of tannin. ‘Crimson Pearl’ and ‘Petite Pearl’ could be used to produce wines with more balanced acidity and address consumer demand for lower-alcohol wines.

The difference between juice IRP and tannin concentrations between vintages at 1 wk PFS may have resulted from higher phenolic content in skins due to greater water availability leading up to that time point in 2022 (399 mm) compared to 2021 (119 mm). García-Esparza et al. [19] described similar results in their study about deficit irrigation regimes on *V. vinifera* cv. ‘Cabernet Sauvignon’. Additionally, since the seeds are soft early in development [10], the higher rainfall might have caused seeds to be even softer in 2022 than in 2021, allowing for greater extraction from seeds to juice during processing.

The large variation in IRP and tannin contents between cultivars compared to contents observed in seeds might be explained by the inconsistent particle size of skins. Further, increased sunlight exposure has been shown to increase the content of flavonols, such as quercetin and myricetin, and anthocyanins in skins [20,21,22]. Interestingly, Del-Castillo-Alonso and colleagues [20] observed varied responses to UV exposure depending on the class of phenolic compounds despite no change in the overall levels of phenolic compounds (measured by spectrophotometry) in the skins of *V. vinifera* cv. ‘Tempranillo’. However, the changes observed between fruit set and véraison were not always maintained at harvest [20]. Further, Blank et al. [23] found a greater effect of weather on changes in skin phenolics than in seeds. This indicates, in combination with the results from the current study, that the phenolic content in skins fluctuates throughout development and ripening in response to several factors, such as sampling time in relation to cultivar-dependent phenology and weather events that may increase or decrease sun exposure. The seeds are not exposed to sunlight, which may result in a more regular trend.

On the other hand, anthocyanins have been shown to impact tannin extractability in model wine solutions [24]. After addition at different concentrations of malvidin-3,5-*O*-diglucoside and malvidin-3-*O*-monoglucoside to ‘Sauvignon Blanc’ must, Campbell et al. [24] noted that the monoglucoside form reacted faster with condensed tannins than the diglucoside form to form polymeric pigments. It is possible that differences in anthocyanin structure and concentration were factors in differences in IRP and tannin extraction observed between the hybrid cultivars and ‘Pinot noir’ in this study. 

Despite different trends throughout development and ripening, skin IRP (6 to 25 mg/g dry skin) and tannin (5 to 12 mg/g dry skin) contents at harvest were comparable between years for each cultivar. Different developmental patterns in skin and seed tannin contents between growing seasons with nearly equivalent contents at harvest for each year have been previously described in *V. vinifera* cv. ‘Cabernet Sauvignon’ [9]. Specifically, the skin and seed tannin contents were about the same at harvest, but the values converged at different times during ripening (i.e., at four weeks prior to harvest in 1998 and harvest maturity in 1999) [9]. Similar differences in skin and seed tannin contents were observed by Cheng et al. [13] in ‘Marquette’ over two growing seasons. In 2020, the tannin content of the skins and seeds was in the same range (<10 mg/g), whereas in 2021, the seeds showed tannin content two to ten times higher (15 to 80 mg/g) than that of the skins (<10 mg/g). The results of the current study showed a trend in both years of phenolic content in skins and seeds decreasing from the 1 wk PFS time point until just before véraison, followed by an increase around véraison and subsequent decrease until harvest (Figure 3 and Figure 4), as previously observed [9,11,13]. Changes in tannin content related to berry maturity have been previously described by Bindon et al. [25]. That study detailed higher molecular mass tannins that became unextractable with increased ripeness. The change in extractability was attributed to changes in cell wall porosity because of degradation of the middle lamella, thus increasing surface area for tannin binding [25]. Any differences between years observed in the current study could be related to differences in extract dissolution procedure or weather patterns resulting in changes to cell wall material. Changes to cell wall material could affect the solubility of extracts, which relates to the modified extract dissolution procedure adapted in 2022. Figure A2 shows a comparison of extract dissolution methods and demonstrates that regardless of the method used, differences are apparent between vintages, especially at 1 wk PFS.

Seed IRP and tannin contents were lower in hybrids (13 to 245 mg IRP/g dry seed and 4 to 171 mg tannin/g dry seed) than in ‘Pinot noir’ (119 to 361 mg IRP/g dry seed and 75 to 384 mg tannin/g dry seed) at each phenological time point in 2022. Our results indicate that this difference between species is probably due to less biosynthesis in seeds, as the same extraction solvent was used for hybrids and for ‘Pinot noir’. However, more work on gene expression, on the one hand, and extractability, on the other, is needed to confirm this assumption. Although the vine trellis system has been shown to modify total phenolics per berry and precipitable tannin from seeds extracts, no difference in total phenols was observed for either skin or seed extracts [26]. This supports the current results indicating differences in seed phenolic content between the hybrids and ‘Pinot noir’ despite different trellising systems used between locations.

**Figure A1:**
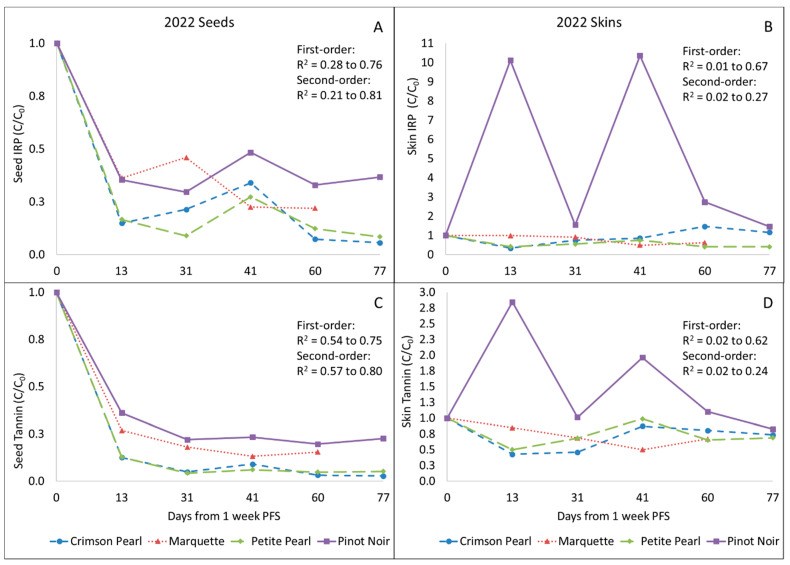
Normalized seed iron-reactive phenolics (IRP) (**A**), skin IRP (**B**), seed tannin (**C**), skin tannin (**D**): content (*C*) at six phenological time points (days from 1 week post-fruit set [PFS]) divided by content at day 0 (*C*_0_) of four grape cultivars throughout development and ripening in 2022.

**Figure A2:**
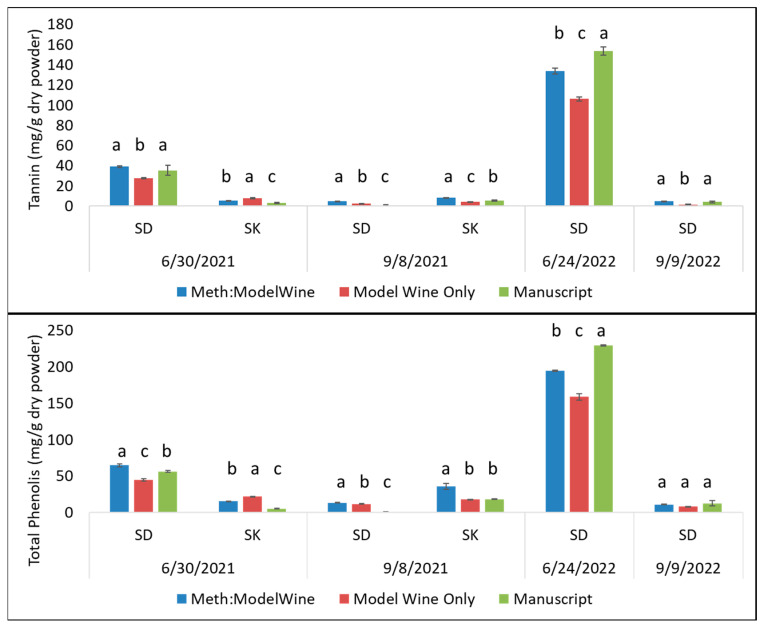
‘Crimson Pearl’ skin/seed extract dissolution method comparison. Different letters indicate statistical differences (*p* < 0.05) between bars within the same date and tissue type.

Interestingly, after normalization, ‘Crimson Pearl’ and ‘Petite Pearl’ showed a greater decrease in seed IRP content than both ‘Pinot noir’ and ‘Marquette’ between 1 wk PFS and later time points (Figure A1A). The variation in IRP may be explained by differences in cell material that limit phenolics extraction. Since ‘Marquette’ has ‘Pinot noir’ in its pedigree, the cell structural material may be more alike compared to ‘Crimson Pearl’ and ‘Petite Pearl’, which are crosses from the same parent vines. Also, after normalization, a greater decrease in seed tannin content was observed for all three hybrid grape cultivars compared to ‘Pinot noir’ for the same time period (Figure A1C). The difference between hybrids and ‘Pinot noir’ could be because ‘Pinot noir’ was grown in a different location (i.e., Michigan), which means different weather patterns could lead to a different timing and length of each phenological time point and phenolics accumulation. For example, Blank et al. [23] found tannin synthesis was correlated to warmer conditions between flowering and véraison for ‘Pinot noir’ over 11 vintages. However, more GDD (i.e., warmer conditions) were accumulated in Iowa (hybrids) than in Michigan (‘Pinot noir’) and at a faster rate over the entire 2022 growing season. This demonstrates that there is an underlying cause for the disparity in IRP and tannin contents between the cold-hardy hybrids and ‘Pinot noir’, possibly related to gene regulation and the expression of tannins. After normalizing IRP and tannin contents in the skins of ‘Marquette’, a steadier decline throughout development and ripening than ‘Crimson Pearl’ or ‘Petite Pearl’ was observed, ultimately decreasing to around 65% of the initial content. In contrast, ‘Crimson Pearl’ and ‘Petite Pearl’ showed a steeper decline in skin IRP and tannin contents from 1 wk PFS to 3 wks PFS but then a greater recovery than ‘Marquette’ around véraison. First- and second-order rate Equations (A1) and (A2) were investigated to explain the net decrease in phenolics observed from 1 wk PFS to harvest, but neither model was able to explain the change (as noted by low R^2^ values, Figure A1), which indicated a more complex dynamic during development and ripening.

## 5. Conclusions

Insight into the basic chemical parameters and phenolic content of understudied grape cultivars is valuable information for grape growers and winemakers to produce the best-quality wine possible. Lower acidity and °Brix in ‘Crimson Pearl’ and ‘Petite Pearl’ would allow winemakers to produce more balanced wines and address growing consumer demand for lower-alcohol wines. This is the first study examining the phenolics and tannin contents of ‘Crimson Pearl’ and ‘Petite Pearl’ cultivars throughout berry development and ripening. It is still uncertain whether the underlying cause of lower tannin content than *V. vinifera* is a result of (i) enzymatic oxidation, due to different polyphenol oxidase activity in hybrid cultivars, (ii) different biosynthesis, which will be investigated further, or (iii) poorer extraction because of inherent differences in cell wall material between species. Future research on extractability would include an investigation into the tannin-binding affinity to the cell wall polysaccharides and proteins of these cultivars.

## Figures and Tables

**Table 1:** Basic chemical properties of grape juices at each phenological time point during berry development and ripening.

Vintage	Variety	Developmental Stage	°Brix		pH		TA ^a^ (g/L)	
2021	‘Crimson Pearl’	1 week PFS ^bc^	3.7 ± 0.2	E ^e^ b ^f^	2.49 ± 0.01	D b	32.1	B a
		3 weeks PFS ^c^	4.2 ± 0.0	D b	2.39 ± 0.01	E b	38.0	A a
		Véraison ^c^	13.3 ± 0.1	C b	2.62 ± 0.01	C a	18.9	C a
		Mid-ripening ^c^	19.0 ± 0.0	B b	3.10 ± 0.01	B a	8.3	D b
		Harvest ^d^	21.3 ± 0.0	A b	3.32 ± 0.01	A a	5.1	E c
	‘Marquette’	1 week PFS ^c^	4.7 ± 0.0	D a	2.60 ± 0.00	C a	26.3 ± 0.1	B a
		3 weeks PFS ^c^	4.9 ± 0.0	D a	2.53 ± 0.01	D a	36.3 ± 0.6	A a
		Véraison ^c^	16.7 ± 0.4	C a	2.54 ± 0.01	D b	24.5 ± 0.4	C a
		Mid-ripening ^c^	22.6 ± 0.2	B a	2.90 ± 0.01	B b	18.0 ± 0.2	D a
		Harvest ^d^	26.7 ± 0.1	A a	3.01 ± 0.02	A c	10.3 ± 0.0	E a
	‘Petite Pearl’	1 week PFS ^c^	3.5 ± 0.0	D b	2.47 ± 0.01	D b	25.9	AB b
		3 weeks PFS ^c^	3.7 ± 0.1	D c	2.40 ± 0.01	E b	33.5	A a
		Véraison ^c^	7.9 ± 0.1	C c	2.54 ± 0.01	C b	22.4	AB a
		Mid-ripening ^c^	14.6 ± 0.1	B c	2.90 ± 0.01	B b	10.8	BC b
		Harvest ^d^	16.8 ± 0.0	A c	3.12 ± 0.01	A b	7.4 ± 0.4	C b
2022	‘Crimson Pearl’	1 week PFS ^c^	4.4 ± 0.0	D c	2.49 ± 0.24	B a	25.6 ± 0.6	B a
		3 weeks PFS ^c^	3.5 ± 0.1	E b	2.45 ± 0.02	B b	33.6 ± 0.2	A a
		Véraison ^c^	9.9 ± 0.1	C b	2.50 ± 0.03	B b	33.4 ± 0.5	A b
		Mid-ripening ^c^	16.3 ± 0.0	B c	2.94 ± 0.01	A b	13.1 ± 0.1	C b
		Harvest ^d^	19.1 ± 0.1	A b	3.18 ± 0.02	A a	8.1 ± 0.3	D b
	‘Marquette’	1 week PFS ^c^	4.5 ± 0.0	D c	2.64 ± 0.01	C a	23.4 ± 0.0	C b
		3 weeks PFS ^c^	4.0 ± 0.1	D a	2.50 ± 0.02	D a	33.6 ± 0.8	B a
		Véraison ^c^	7.7 ± 0.0	C c	2.59 ± 0.02	CD a	36.4 ± 0.3	A a
		Mid-ripening ^c^	18.6 ± 0.3	B a	2.81 ± 0.01	B d	22.2 ± 0.7	C a
		Harvest ^d^	22.3 ± 1.4	A a	2.99 ± 0.08	A b	13.6 ± 1.1	D a
	‘Petite Pearl’	1 week PFS ^c^	5.2 ± 0.0	D b	2.69 ± 0.02	C a	20.9 ± 0.3	C c
		3 weeks PFS ^c^	3.5 ± 0.0	E b	2.44 ± 0.02	D b	31.0 ± 0.4	A b
		Véraison ^c^	7.7 ± 0.0	C c	2.44 ± 0.00	D c	26.6 ± 0.3	B c
		Mid-ripening ^c^	14.8 ± 0.1	B d	2.84 ± 0.02	B c	12.6 ± 0.2	D b
		Harvest ^d^	19.0 ± 0.1	A b	3.15 ± 0.02	A a	8.2 ± 0.2	E b
	‘Pinot noir’	1 week PFS ^c^	6.2 ± 0.2	D a	2.47 ± 0.01	D a	16.1 ± 0.4	C d
		3 weeks PFS ^c^	3.4 ± 0.1	E b	2.43 ± 0.00	E b	29.7 ± 0.8	A b
		Véraison ^c^	10.2 ± 0.0	C a	2.52 ± 0.00	C b	27.7 ± 0.5	B c
		Mid-ripening ^c^	16.9 ± 0.0	B b	2.99 ± 0.01	B a	12.3 ± 0.2	D b
		Harvest ^d^	21.4 ± 0.1	A a	3.22 ± 0.02	A a	8.2 ± 0.5	E b

^a^ TA: titratable acidity. ^b^ PFS: post-fruit set. ^c^ Values are means of analytical triplicate ± standard deviation. ^d^ Values are means of biological triplicate ± standard deviation. ^e^ Uppercase letters indicate differences (*p* < 0.05) between time points for the same cultivar in each vintage. ^f^ Lowercase letters indicate differences (*p* < 0.05) between cultivars for the same time point in each vintage.

## Data Availability

The original contributions presented in the study are included in the article, further inquiries can be directed to the corresponding author.

## Appendix A

**Table A1:** Sampling dates for each phenological time point.

Vintage	Cultivar	1 Wk PFS ^a^	3 Wks PFS	5 Wks PFS	Véraison	Mid-Ripening	Harvest
2021	‘Crimson Pearl’	30 June	7 July	22 July	4 August	25 August	8 September
	‘Marquette’	22 June	-	-	28 July	11 August	25 August
	‘Petite Pearl’	30 June	7 July	22 July	4 August	25 August	8 September
2022	‘Crimson Pearl’	24 June	7 July	25 July	4 August	23 August	9 September
	‘Marquette’	24 June	7 July	-	25 July	9 August	26 August
	‘Petite Pearl’	24 June	7 July	25 July	4 August	23 August	9 September
	‘Pinot noir’	7 July	21 July	8 August	22 August	7 September	7 October

^a^ PFS: post-fruit set.

**Table A2:** Berry diameter and berry weight throughout the development and ripening of the four cultivars in 2021 and 2022.

Vintage	Cultivar	Developmental Stage	Berry Weight (g) ^d^	Berry Diameter (mm)
2021	‘Crimson Pearl’	1 wk PFS ^ab^	0.56	10.6 ± 0.8 C^e^ a^f^
		3 wks PFS ^b^	0.92	12.3 ± 1.1 B a
		Véraison ^b^	1.37	14.0 ± 1.5 A a
		Mid-ripening ^b^	1.66	13.8 ± 1.1 A a
		Harvest ^c^	1.73	13.9 ± 0.8 A a
	‘Marquette’	1 wk PFS ^b^	0.46	9.0 ± 1.2 C b
		3 wks PFS ^b^	0.77	11.3 ± 1.2 B b
		Véraison ^b^	1.06	12.6 ± 1.2 A b
		Mid-ripening ^b^	1.32	13.4 ± 0.6 A a
		Harvest ^c^	1.40	12.9 ± 1.1 A b
	‘Petite Pearl’	1 wk PFS ^b^	0.41	8.9 ± 0.7 D b
		3 wks PFS ^b^	0.51	9.6 ± 1.3 CD c
		Véraison ^b^	0.57	10.3 ± 0.9 BC c
		Mid-ripening ^b^	0.80	10.6 ± 0.8 AB b
		Harvest ^c^	0.89	11.4 ± 0.7 A c
2022	‘Crimson Pearl’	1 wk PFS ^b^	0.33	8.8 ± 0.6 D a
		3 wks PFS ^b^	0.90	12.2 ± 0.9 C a
		Véraison ^b^	1.27	12.4 ± 1.1 BC a
		Mid-ripening ^b^	1.53	13.3 ± 1.6 AB a
		Harvest ^c^	1.61	13.6 ± 1.2 A a
	‘Marquette’	1 wk PFS ^b^	0.34	7.8 ± 0.8 D b
		3 wks PFS ^b^	0.56	10.4 ± 0.9 C b
		Véraison ^b^	0.77	11 ± 0.8 BC b
		Mid-ripening ^b^	1.07	11.9 ± 1 AB b
		Harvest ^c^	1.30	12.6 ± 1.3 A b
	‘Petite Pearl’	1 wk PFS ^b^	0.23	7.3 ± 0.5 D b
		3 wks PFS ^b^	0.46	9.8 ± 0.8 C b
		Véraison ^b^	0.72	10.7 ± 1.5 B b
		Mid-ripening ^b^	0.86	10.9 ± 1.1 AB b
		Harvest ^c^	0.93	11.4 ± 0.9 A c
	‘Pinot noir’	1 wk PFS ^b^	0.09	5 ± 0.6 D c
		3 wks PFS ^b^	0.46	8.7 ± 1.1 C c
		Véraison ^b^	1.00	11.1 ± 1.3 B b
		Mid-ripening ^b^	0.99	11.2 ± 1.2 B b
		Harvest ^c^	1.55	13.6 ± 1.3 A a

^a^ PFS: post-fruit set. ^b^ Values are means of analytical triplicate ± standard deviation. ^c^ Values are means of biological triplicate ± standard deviation. ^d^ Berry weight was calculated by dividing the weight of 50 berries by 50. ^e^ Uppercase letters indicate differences (*p* < 0.05) between time points for the same cultivar in each vintage. ^f^ Lowercase letters indicate differences (*p* < 0.05) between cultivars for the same time point in each vintage.

First- and second-order rate equations were fit to the normalized skin and seed IRP and tannin contents according to Equations (A1) and (A2).

[*C*] = [*C*]_0_e^−*kt*^ (A1)

1/[*C*] = 1/[*C*]_0_ + *kt* (A2)

Figure A2 shows a comparison of a subset of ‘Crimson Pearl’ skin and seed extracts (70% acetone, as detailed in Section 2.6) that were solubilized using both dissolution methods compared to values reported in the main text of this manuscript (Figure 3 and Figure 4). “Meth:ModelWine” denotes extracts dissolved using the modified 2022 method, “Model Wine Only” denotes extracts dissolved using the 2021 method, and “Manuscript” denotes values reported in Figure 3 and Figure 4.
